# Effective Management of Refractory Hepatic Hydrothorax With Hypertonic Glucose Pleurodesis

**DOI:** 10.7759/cureus.81083

**Published:** 2025-03-24

**Authors:** Shuya Hashimoto, Toyoshi Yanagihara, Natsumi Kushima, Rei Sanai, Takato Ikeda, Naoki Hamada, Masaki Fujita

**Affiliations:** 1 Department of Respiratory Medicine, Fukuoka University Hospital, Fukuoka, JPN

**Keywords:** dyspnea, hepatic hydrothorax, hypertonic glucose, liver cirrhosis, pleurodesis

## Abstract

Hepatic hydrothorax presents a significant challenge in advanced liver cirrhosis management, especially when conventional therapies fail. We report a case of a 66-year-old male with hepatitis C-related cirrhosis and advanced hepatocellular carcinoma who developed refractory left-sided hepatic hydrothorax despite maximal diuretic therapy. After failed talc pleurodesis, we employed an alternative approach using 50% glucose solution as a sclerosing agent. Following three sessions of hypertonic glucose pleurodesis, the patient achieved sustained resolution of pleural effusion with only minor, transient complications including hypotension and mild inflammatory response. Notably, he did not develop significant renal dysfunction or hepatic decompensation, complications commonly associated with conventional sclerosing agents in liver cirrhosis. Two-month follow-up imaging confirmed persistent pleural adhesion. This case highlights hypertonic glucose pleurodesis as a potentially safer alternative for managing refractory hepatic hydrothorax, particularly in patients unsuitable for transjugular intrahepatic portosystemic shunt or liver transplantation. However, further research is needed to establish optimal protocols, patient selection criteria, and long-term outcomes of this approach.

## Introduction

Hepatic hydrothorax is a rare but challenging complication of liver cirrhosis, occurring in approximately 5%-10% of patients with advanced disease of liver cirrhosis [1,2]. It results from the transdiaphragmatic movement of ascitic fluid into the pleural cavity due to increased intra-abdominal pressure and diaphragmatic defects [3]. Unlike ascites, hepatic hydrothorax can lead to significant respiratory distress even with a relatively small volume of pleural effusion. Conventional management includes sodium restriction, diuretics, and therapeutic thoracentesis, while more advanced cases may require trans-jugular intrahepatic portosystemic shunt (TIPS) or liver transplantation [3]. However, these options are not always feasible due to contraindications, procedural risks, or limited availability.

Pleurodesis has been attempted as a palliative intervention in refractory cases of hepatic hydrothorax. Various sclerosing agents, including talc, tetracycline, and povidone-iodine, have been used, but their efficacy is inconsistent, and they carry risks such as systemic inflammation, renal dysfunction, and worsening hepatic failure [4,5]. Recently, hypertonic glucose has been proposed as an alternative pleurodesis agent due to its ability to induce pleural adhesion with potentially fewer systemic complications [6]. However, no reports exist supporting its effectiveness in hepatic hydrothorax. Here, we present a case of successful pleurodesis using hypertonic glucose in a patient with refractory hepatic hydrothorax, highlighting its potential role and the need for further investigation.

## Case presentation

A 66-year-old male with liver cirrhosis secondary to hepatitis C and Stage IVB poorly differentiated hepatocellular carcinoma presented with progressive exertional dyspnea. His medical history was significant for left diaphragmatic metastasis resection and treatment with Bevacizumab and Atezolizumab. Despite maximal diuretic therapy, he experienced recurrent left pleural effusion requiring frequent thoracentesis. The patient had previously achieved hepatitis C viral clearance with ombitasvir/paritaprevir/ritonavir therapy but subsequently developed hepatocellular carcinoma with lymph node metastasis. He had undergone partial hepatectomy (S8/5) and resection of a left diaphragmatic tumor with mesh repair. At presentation, he was classified as Child-Pugh grade B (8-9 points) with comorbidities including esophageal varices, portal vein thrombosis, hypertension, and arrhythmia.

Following his surgical intervention, the patient developed persistent left-sided pleural effusion refractory to optimized diuretic therapy (spironolactone, azosemide, tolvaptan). Four months prior to the current presentation, his condition deteriorated, necessitating emergency hospitalization for acute respiratory distress. Pleural fluid analysis revealed a transudate (total protein 1.7 g/dL, LDH 57 U/L, glucose 107 mg/dL) with serum values showing total protein of 6.9 g/dL and LDH of 167 U/L, yielding a pleural fluid-to-serum protein ratio of 0.25 and LDH ratio of 0.34. Cell count demonstrated 68 cells/μL with a lymphocyte predominance (45%), and cytological examination showed no malignant cells. Bacterial and fungal cultures were negative, consistent with non-infected hepatic hydrothorax.

Three months prior, conventional talc pleurodesis was attempted following left thoracostomy drainage, but the pleural effusion recurred within weeks. Due to persistent fluid accumulation, an alternative approach using hypertonic glucose pleurodesis was initiated two months prior. A 20Fr double-lumen chest tube was inserted, and 200 mL of sterile 50% glucose solution was instilled into the pleural cavity via the chest tube. The chest tube was clamped during instillation. Pleural drainage was resumed two hours after the glucose solution instillation. During the procedure, we continuously monitored vital signs (blood pressure, heart rate, respiratory rate, and oxygen saturation), subjective symptoms (cough, chest discomfort, pain using a visual analog scale, and degree of dyspnea), and blood glucose levels. This procedure resulted in temporary clinical improvement, with the patient experiencing significant relief of dyspnea and radiographic evidence of decreased pleural effusion volume, allowing for hospital discharge.

**Figure 1:**
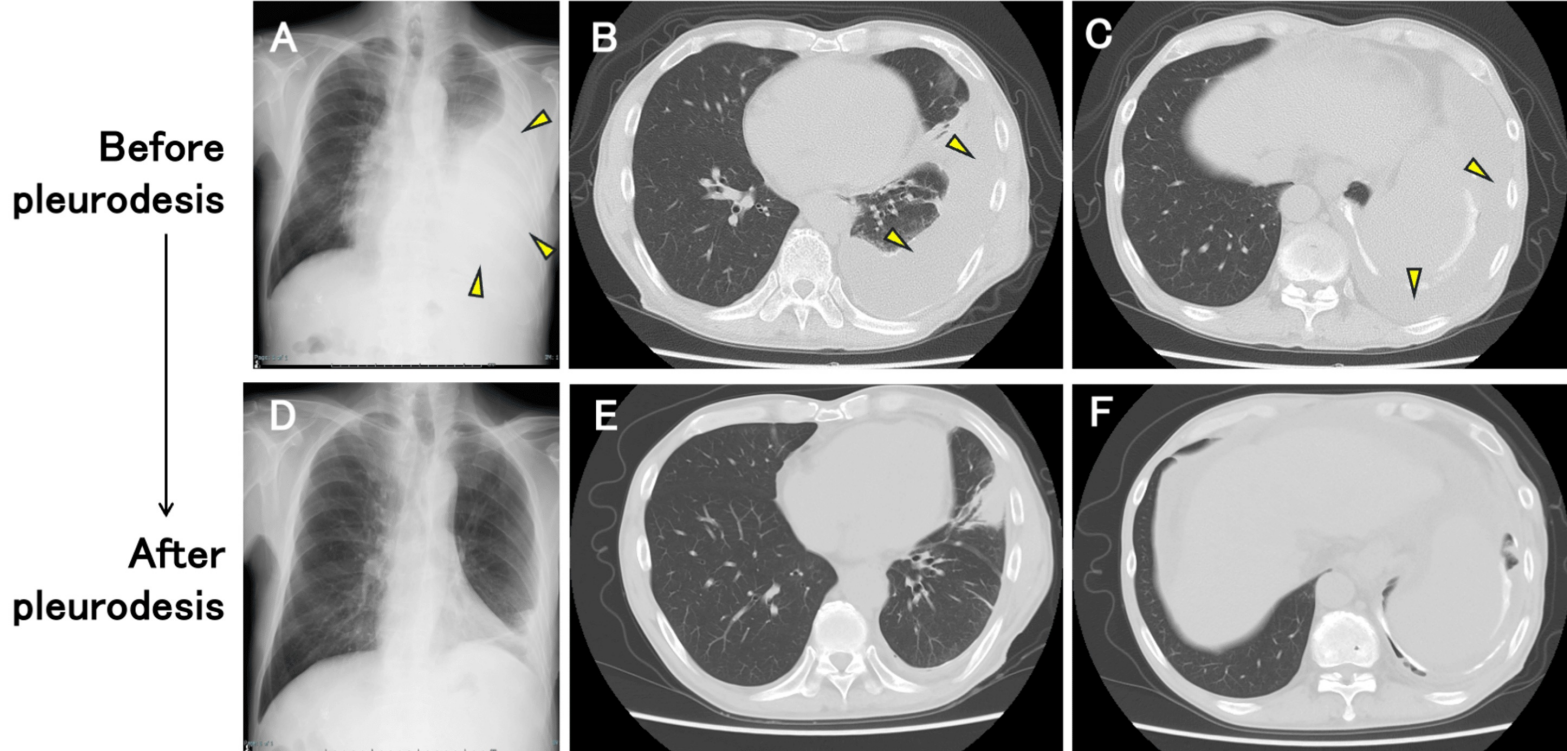
Radiographic findings before and after hypertonic glucose pleurodesis in a patient with refractory hepatic hydrothorax. (A-C) Images before pleurodesis showing significant left-sided pleural effusion. (A) Chest radiograph demonstrating large left pleural effusion with partial compression of the left lung. (B, C) Axial CT images showing extensive left pleural effusion with compressive atelectasis and mediastinal shift. Arrowheads indicate pleural effusion. (D-F) Images after completion of hypertonic glucose pleurodesis. (D) Chest radiograph showing significant resolution of the left pleural effusion. (E, F) Axial CT images demonstrating successful pleural adhesion with near-complete resolution of the pleural effusion and re-expansion of the left lung.

Despite initial improvement, pleural fluid reaccumulated within one month (Figures 1A-1C), requiring multiple thoracenteses. The patient was readmitted for definitive management. During this hospitalization, a second session of 50% glucose pleurodesis was performed on day 3, followed by a third session on day 10. The patient experienced only minor, self-limiting complications during the procedures. These included transient hypotension (baseline blood pressure of 90/50 mmHg decreasing to 60-80/40 mmHg temporarily, which recovered without additional fluid administration) and a mild inflammatory response (transient fever up to 38°C and elevated C-reactive protein levels up to 8 mg/dL, comparable to his previous reaction to talc pleurodesis). Notably, he did not develop renal failure, systemic infection, or severe metabolic derangements. The chest drain was removed on day 13, and the patient was discharged on day 16. At the two-month follow-up, imaging studies confirmed persistent pleural adhesion with no recurrence of pleural effusion (Figures 1D-1F). Interestingly, increased ascites were observed, suggesting successful prevention of transdiaphragmatic fluid migration.

## Discussion

Managing refractory hepatic hydrothorax remains a significant clinical challenge, particularly when standard treatments fail or are contraindicated [4]. Repeated thoracentesis provides temporary relief but poses risks including pneumothorax, protein depletion, and infection. TIPS effectively reduces ascites and pleural effusion by decreasing portal pressure, yet it is associated with hepatic encephalopathy and may be contraindicated in patients with advanced liver dysfunction [7]. Liver transplantation remains the definitive treatment but is often unavailable due to donor shortages and patient eligibility constraints [4].

Pleurodesis is generally not considered a first-line treatment for hepatic hydrothorax due to concerns regarding its safety and efficacy in cirrhotic patients [4]. Previous studies have shown mixed results, with chemical pleurodesis (OK-432, talc, tetracycline, minocycline, etc.) achieving success rates of approximately 60%-80%, but often with significant adverse events [8]. The literature reports complications in up to 82% of patients receiving inflammatory sclerosing agents, including renal failure (17.5%), hepatic encephalopathy (11%), pneumonia (9.5%), and sepsis (3%) [8]. Another study reported severe complications including acute renal failure and hepatic encephalopathy with a 45% mortality rate [5].

In our case, initial talc pleurodesis failed to achieve adequate pleural adhesion. Given the documented risk of serious complications with conventional sclerosing agents such as OK-432 or minocycline, we reconsidered our approach and selected 50% glucose solution as an alternative. Hypertonic glucose pleurodesis for pneumothorax is described in the European Respiratory Society task force statement. Studies examining its efficacy in secondary spontaneous pneumothorax have reported success rates comparable to minocycline and talc [6,9]. To our knowledge, this is the first English-language report of hypertonic glucose pleurodesis for hepatic hydrothorax. Several factors may have contributed to the success observed in our case. First, the ability to safely administer repeated applications of 50% glucose solution provided multiple opportunities for pleural adhesion formation. Second, the relatively large volume (200 mL) of sclerosing agent used may have been advantageous. In hepatic hydrothorax, rapid fluid accumulation can dilute sclerosing agents during drain clamping periods, potentially reducing their effectiveness. The substantial volume of 50% glucose solution employed may have better maintained an effective concentration despite this dilution effect, enhancing the likelihood of successful pleurodesis.

Hypertonic glucose offers several advantages. It is cost-effective, can be administered repeatedly unlike talc, and may have a lower risk of systemic toxicity compared to other agents such as OK-432 or tetracyclines. However, potential complications include hyperglycemia [10], dehydration (which can lead to ischemic colitis) [11], renal dysfunction, and acute respiratory distress syndrome (ARDS) leading to a fatal outcome [12]. Cirrhotic patients are particularly vulnerable to hemodynamic instability, as evidenced by the transient hypotension observed in our patients. When employing this technique, regular monitoring of blood pressure is essential, with consideration for prophylactic fluid administration if needed.

Another safe alternative is autologous blood patch pleurodesis, commonly used for air leak cessation in pneumothorax [13]. However, this technique typically requires more than 100 mL of blood, making it potentially unsafe for anemic patients with liver cirrhosis.

Our case adds to the growing body of evidence suggesting that hypertonic glucose pleurodesis may be beneficial for hepatic hydrothorax in carefully selected patients. However, further studies, including prospective trials and larger case series, are needed to establish its safety, efficacy, and long-term outcomes. These studies may guide the personalized optimal protocols for managing hepatic hydrothorax as indicated by the liver function and frail condition. Until then, its use should be considered on a case-by-case basis, with close monitoring for potential complications. Future research should focus on identifying optimal patient selection criteria, determining the ideal glucose concentration and volume, and establishing standardized protocols to maximize efficacy while minimizing adverse events.

## Conclusions

In conclusion, hypertonic glucose pleurodesis presents a promising alternative for managing refractory hepatic hydrothorax, especially in patients for whom conventional treatments such as TIPS or liver transplantation are not viable. While our case demonstrated successful pleurodesis without severe complications, the overall safety and efficacy of hypertonic glucose in this setting remain to be confirmed. Given the potential risks of transient hypotension and fluid shifts, careful patient selection, regular monitoring, and further clinical studies are warranted to better define the role of hypertonic glucose pleurodesis in hepatic hydrothorax management.
